# Does maternal overnutrition carry child undernutrition in India?

**DOI:** 10.1371/journal.pone.0265788

**Published:** 2022-06-17

**Authors:** Mukesh Kumar, Pratap Mohanty

**Affiliations:** Department of Humanities and Social Sciences, Indian Institute of Technology Roorkee, Roorkee, Uttarakhand, India; Texas A&M University College Station, UNITED STATES

## Abstract

**Background and objectives:**

Studies in low-and middle-income countries where nutrition transition is underway provides mixed evidence of double burden of maternal overnutrition and child undernutrition among mother-child pairs. Shifting dietary pattern and rapid increase in overweight/obesity among adults with persistent child undernutrition indicate that India is experiencing nutrition transition and double burden of malnutrition. Hence, the study explores the presence of and the factors associated with mother-child dyads of over- and undernutrition in India.

**Methods and materials:**

The study uses National Family Health Survey 2015–16 data. The analytic sample consists of 28,817 weighted mother-child pairs where an overweight/obese mother is paired with an undernourished child. The nutritional status of children is defined according to WHO 2006 child growth standards as underweight (i.e., low weight-for-age), stunting (i.e., low height-for-age) and wasting (i.e., low weight-for-height). Maternal overweight/obesity (i.e., BMI ≥ 25 kg/m^2^) is defined using adult BMI criterion. Descriptive, bivariate, and adjusted multivariable logistic regression analysis are conducted.

**Results:**

Of the overweight/obese mothers, 21.3%, 26.5%, and 14% have underweight, stunted, and wasted children respectively. In adjusted models, maternal short stature (aOR: 2.94, 95% CI: 2.30–3.75), age of child (aOR: 3.29, 95% CI: 2.76–3.92), and poorest wealth status (aOR: 2.01, 95% CI: 1.59–2.54) are significant predictors of overweight/obese mothers and stunted child pairs. Similarly, poor wealth status (aOR: 1.68, 95% CI:1.32–2.14), maternal stature (aOR: 2.70, 95% CI: 2.08–3.52), and child aged 2–5 years (aOR: 1.77, 95% CI:1.51–2.08) are also significantly associated with higher occurrence of overweight/obese mother and-underweight child pairs.

**Conclusion:**

Findings of the study are consistent with the phase of nutrition transition and double burden of malnutrition. The paper concludes with suggestions to improve the socioeconomic condition, more strategic nutrition specific investments and policy interventions to eliminate all forms of malnutrition for achieving SDGs.

## 1. Introduction

The global nutrition report of 2017 highlighted that “*a better-nourished world is a better world”* [1]. Malnutrition is one of the biggest threats to human and economic progress and responsible for adverse health condition than any other causes [2, 3]. According to the recent estimates of child malnutrition, 47 (6.9%) million children under-five years of age suffer from wasting and almost 144 (21.3%) million are stunted, and 38.3 (5.6%) million are overweight [4]. Every nation in the world is witnessing some form of nutritional crisis [5]. Progress towards reducing the undernourishment is unacceptably far slow and unequal across the globe, regions, and within a country [6, 7].

Undernutrition is still a major challenge for low-and middle-income countries (LMICs) while High-Income Countries (HICs) are facing the other side, a rapid surge in overweight/obesity [8]. Globally, in 2019, Food and Agriculture Organisation (FAO) estimated that 821.8 million people are undernourished and the number is on the rise since 2015. Moreover, in 2019, approximately one-third of total population in the world was overweight/obese and the number amounts to 2.28 billion children and adults [9, 10].

Maternal and child malnutrition is responsible for more than four (4.5) out of ten under-five children deaths, globally, and overweight/obesity causes four million deaths annually which is around five percent of the total deaths [9–11]. Malnutrition is linked throughout the life cycle, and an early age growth faltering and rapid weight gain may lead to excess weight and other risks in the adulthood [12, 13]. Undernutrition in foetal and childhood contributes to both immediate (such as stunted physical and cognitive achievement) and long-term ill-health leading to cardiometabolic diseases (diabetes, hypertension, cardiovascular disease) [14]. Malnutrition also increases economic costs in the form of higher expenditure on healthcare and loss of human capital. An estimate shows that undernutrition reduces the Gross Domestic Product (GDP) about 11 percent in Asia and Africa [15]. Similarly, overweight/obesity has lasting personal, social, and economic impacts, and the annual cost of obesity was projected to be about US$ 2 trillion in 2014, globally [16]. Due to limitations of modelling issue, nutrition economists estimate economic and health costs of under-and-overnutrition separately and add them to find the combined burdens of malnutrition [17].

Malnutrition may coexist in multiple forms in a country depending upon its phase of the *nutrition transition*. [18, 19]. According to Barry M. Popkin, the concept of nutrition transition largely focuses on the shift in composition and structure of diet and activity patterns in five stages. This structural shift in dietary and activity patterns reflects in nutritional outcomes, health status, and socioeconomic and demographic changes [19]. Based on Popkin’s nutrition transition patterns, India seems to be entered fourth stage—degenerative disease—that is characterised by large proportion of fat in food intake, increase in adult obesity, decline in fertility rate, diet related chronic diseases, and rapid growth in income and income disparities.

Many LMICs, where the nutrition transition is underway, are experiencing a rapid increase in obesity among adult women irrespective of their place of residence even with a persistently high prevalence of child malnutrition [8, 20]. The double burden of malnutrition (undernutrition as well as overweight/obesity in the same setting) is two sides of the same nutritional crisis and more often occurs in LMICs due to nutrition disparities [21]. Double Burden of Malnutrition (DBM hereafter) is an operational concept and refers to the coexistence of undernutrition as well as overweight/obesity at population, household, dyad, or individual level. A recent study by Davis and colleagues (2020) provides a systematic review of operational definitions of DBM and concludes that overweight/obesity with underweight, thinness, stunting, or wasting are most studied form double burden of malnutrition [22]. To study the intrahousehold DBM, researchers consider the mother-child dyad as a unit of analysis. The mother child dyad framework, which we adopt in present study, defines mother-child level double burden of malnutrition (MCDBM) as the presence of an overweight/obese mother and at least one undernourished child in the same household [8, 23, 24].

Due to its profound public health policy and economic implications DBM is studied widely across the world [8, 25–28]. Previously, some studies in LMICs have explored the MCDBM at the household level by using mother-child pair (overweight/obese mother with undernourished child) as a unit of analysis and find substantial differences in prevalence. The prevalence of MCDBM was 19.6% in Kenya [23], 11% in South Africa [29], and 5.1% in Colombia [30]. Asia and the Pacific countries are the most prominent economies over the last 20 years and many of them are experiencing the nutrition transition that results in coexistence of under-and overnutrition/DBM [31, 32].

In last two decades, India experienced much better rates of economic growth and changing food preferences which reflects that the nutrition transition is underway [33, 34]. Unfortunately, despite a high growth rate child malnutrition is not reduced significantly and 35%, 33%, and 17% of children under-five years of age are stunted, underweight, and wasted, respectively [35]. The incidence of overweight/obesity has increased unexpectedly in poorer and populous states in India [36]. Furthermore, this may give rise to the MCDBM in poor settings. In India, only the DBM—not the MCDBM—is explored among adults [37], reproductive-age women [38], and adolescents [39] at population and specific region level [40, 41] but none of these studies considered the mother-child pair at household level as an unit of analysis.

However, a limited number of studies within a state or at a specific location; [42] in Kerala and [43] in Delhi, have explored the DBM as mother-child dyads. The presence of DBM is likely to occur in India as corroborated by the co-occurrence of high child malnutrition and a dramatic rise in overweight/obesity among adults. Therefore, this study aims to quantify the DBM in mother-child dyads and to identify the key socioeconomic and demographic determinants in India. Findings of the study are expected to help nutritionists and policymakers to design the nutrition-related interventions which are crucial to eliminate all forms of malnutrition and to fulfil the Sustainable Development Goals (SDG) by 2030.

The remaining paper proceeds as follows: Section 2 describes the survey population, survey implementation and sampling technique, variables included in the study and their measurement, and data analysis technique. Section 3 documents the interpretations of results. Section 4 discusses the findings, policy implications and limitations of the study. Finally, section 5 presents the conclusion of the study.

## 2. Methods

### 2.1. Study design and population

The present study utilises data from the most recently available nationally representative household survey, National Family Health Survey round four (NFHS-4), conducted from January 2015 to December 2016 [44]. The NFHS-4 was implemented by the International Institute of Population Sciences (IIPS) Mumbai under the guidance of the Ministry of Health and Family Welfare (MoHEW). The survey was supported by the Inner City Fund International, Maryland, and the United States Agency for International Development (USAID). NFHS-4 collected information on key population, health, socioeconomic, and anthropometric measures. For the first time, NFHS-4 extended its scope to all the states (29) and union territories (7) as well as 640 districts and provided information on key indicators separately for urban and rural areas. It is reported that NFHS surveys cover 99% population of India [45]. Master Sampling framework for the survey was the 2011 Census of India [44]. The study is based on secondary data which anonymizes the particulars of respondents. Therefore, Institutional Review Board (IRB) examination is not applicable in our use of data.

#### 2.1.1. Sample and sampling strategy

NFHS-4 questionnaires were canvassed in the 6,01,509 households (including men aged 15–54 years, women aged 15–49 years, and children under-five year of age) across the country using a two-stage stratified cluster sampling design. After the rural-urban stratification, 28,586 Primary Sampling Units (PSU) were selected in the first stage using a probability proportional to the size sample method within each stratum. PSUs are the census enumeration block in the urban area and villages in rural areas. Out of the 28,586 selected PSUs, fieldwork was completed in 28,522 clusters with a response rate of 98%. In the second stage, based on a complete household listing, a fixed number of 22 households from each rural and urban cluster were selected using a systematic random sampling technique. More details on sampling design and methodology of NFHS-4 is available in the report of NFHS-4 [44].

Among the 6,01,509 households selected for the entire household questionnaire, anthropometric (height and weight) measurements were taken for 6,99,686 eligible (15–49 years) women, 1,24,385 (15–54 years) men, and 2,59,627 children under-five years of age. For the analysis purpose, the present study uses data mainly from child file to construct the mother-child dyads. Child file contains detailed information on anthropometric and health status of children, their mothers, and important household characteristics. Due to some variation in availability of valid background information, our final analytical sample varies between 27,081 to 28,817 weighted mother-child pairs having valid anthropometric measurement and complete sociodemographic information. Further, NFHS does not collect comprehensive information on occupations, therefore occupation related information is available for 5,296 respondents.

#### 2.1.2. Data collection technique/measurement of nutritional status

To examine the nutritional status, anthropometric (height and weight) measurements of the children and adult were taken by trained health investigators. For eligible children aged 0–59 months and adult men and women weight and height were measured with standard measurement instruments (Weight was taken with electronic SECA 874 digital scales to the nearest 100 g. For height measurement, recumbent length for child age 0–24 months using SECA 213 infantometer and standing height for 24–59 months child and adults was measured by using SECA 417 stadiometer with adjustable Shorr measuring boards) in the NFHS-4 [44]. Recorded height and weight of children were converted into age and sex-adjusted standard Z-score using WHO 2006 child growth standard “zscore06” using package in Stata (StataCorp, College Station, TX, USA) [46]. Standardised Z-scores of height-for-age (HAZ), weight-for-age (WAZ), and weight-for-height (WHZ) are the most widely used indicator of child nutritional status. Children having invalid anthropometric measurement are excluded, i.e., height-for-age or weight-for-age Z-score beyond ±6 or weight-for-height Z-score beyond ±5 standard deviations from the WHO growth standard. Stunting, underweight, and wasting are defined as HAZ, WAZ and WHZ score below -2 standard deviation from the WHO 2006 child growth standard. To measure the nutritional status of mothers of eligible children, maternal height and weight were recorded in the survey. Pregnant women at the time of the survey, those who gave birth in the preceding two months from the date of the survey, and those with invalid anthropometric measurement are excluded from the analysis. Maternal nutrition status is defined as per WHO (2006) body mass index (BMI) criteria for adults aged 15–49 and categorised as: underweight (BMI<18.5 kg/m^2^); normal (BMI 18.5–24.99 kg/m^2^); overweight (BMI 25–29.99 kg/m^2^); and obese (BMI>29.99 kg/m^2^). For analysis purpose, we classify overweight/obese women as having BMI>24.99 kg/m^2^.

### 2.2. Study variables

#### 2.2.1. Outcome variables

In the present study, the analysis is limited to the household level DBM where at least one under-five age child is undernourished (stunted/underweight/wasted), and his or her mother is overweight/obese. First, we dichotomised the mother’s nutrition-related outcome variable as: ‘1’ implies the presence of maternal overweight/obesity, ‘0’ otherwise. Second, three categorical variables are constructed for child underweight, stunting, and wasting, where ‘1’ represents malnutrition, and ‘0’ otherwise. Finally, based on these dichotomised variables in first two steps, we conceptualised four forms of mother-child double burden of malnutrition (MCDBM). In it’s all form, the mother was either overweight or obese (OWOBM). Exclusive mother-child pairs of OWOBM with stunted (OWOBM-SC), underweight (OWOBM-UC), or wasted (OWOBM-WC) child is defined as three basic forms of MCDBM. Additionally, to assess any form of MCDBM, an overweight/obese mother is paired with a child having at least one (i.e., stunted or underweight, or wasted) form of malnutrition (OWOBM-SUWC) [23]. All four outcome variables are coded as ‘1’ having presence of MCDBM and ‘0’ otherwise.

#### 2.2.2. Sociodemographic and economic characteristics

In the NFHS-4 household, maternal, and child related sociodemographic characteristics are collected using a validated questionnaire in a face-to-face interview with eligible participants. Following the extant literature that provides various household, socioeconomic, biological, and individual specific variables which are key to understand the DBM; we included these factors in the study [23, 47, 48]. At the household level, place of residence (rural- urban), water and sanitation facility (improved—unimproved), social group (caste and religion), and wealth index are included as independent variables following the literature. As a proxy of living standard, the wealth index is a composite score of ownership of 26 assets and the household’s use of other amenities. While generating wealth index, the principal components analysis (PCA) is used to get the factor score of each asset, and then factor scores are standardised on a normal distribution. With household, individual characteristics of mother and children are also crucial in understanding the double burden [49]. Maternal variables include age, height, education, number of children ever born, currently breastfeeding status, and work status of the concerned mother. Characteristics of child include current age, birth order, health status, and sex of the child.

#### 2.2.3. Data analysis

A total 28,817 pairs of overweight/obese mother and undernourished or normal child are used to understand the prevalence of and factors associated with MCDBM in India. For descriptive statistics, we use frequency distribution of pairs of overweight/obese mother and undernourished child by background characteristics, i.e. household, mother, and child-related factors. Due to categorical (dummy) nature of our outcome variables, bivariate logistic regression model is applied to identify the factors associated with MCDBM. Factors which are statistically significant at 5% level *(p value < 0*.*05)* in bivariate regression are controlled in multivariate logistic regression model to identify the independent predictors. Adjusted odds ratio (aOR) and unadjusted odds ratio (uOR) with confidence interval (CI) of 95% are reported in the results [23]. To check the statistical bias in standard errors, we tested for multicollinearity among independent predictors in multivariate models using variance inflation factors (VIF) with a cut-off level of ‘10’. We do not find the issue of multicollinearity in our analysis. To adjust for the complex sampling design used in DHS surveys, sampling weight is applied in the analysis using ‘Svy’ command in the Stata. All statistical analysis is performed using STATA/SE 15.1 (College Station, TX: StataCorp LLC).

## 3. Results

Sociodemographic characteristics of the respondents play important role in understanding the phenomenon under consideration in any study. Table 1 shows sample distribution of socioeconomic and demographic characteristics of the respondents in our study. Almost half (49.2%) of mother-child pairs is residing in the urban area, and 50.7% are in the rural area. Of the participants, 11.8%, 28.3%, and 33.7% are poorer, richer, and richest respectively in wealth category. About 96.7% of families have access to improved water facilities but only 63.9% of families have improved sanitation facilities in their households. As per the social groups approximately one-half (46.7%) of the participants belong to other-backward class (OBC), and 18.4% and 46.7% are from Scheduled Caste and Others (general caste group). Three-fourth (73.4%) of the participants are Hindu and 20% are Muslims. Average height of the mother is 152.2 (SD 6.35) cm. Mean age of the mother and children is 28.4 (SD 4.90) years and 32.7 (SD 16.6) months. Mean number of the children ever-born is 2.3 (SD 1.3). Almost fifteen percent (14.2%) mothers are illiterate, 40% received secondary education, and only 21.7% are college graduate. More than half (53.4%) are male child. Of the children 12.2%, 13.9%, and 8.1% had reported cough, fever, and diarrhoea in last two weeks before the survey.

**Table 1:** Sociodemographic characteristics of the respondents, NFHS-4 (2015–16), India.

Household Characteristics		Weighted-n	% or mean
**Residence**	Urban	14183	49.22
	Rural	14634	50.78
**Wealth quintile**	Poorest	1822	6.32
	Poorer	3417	11.86
	Middle	5673	19.69
	Richer	8171	28.36
	Richest	9732	33.77
**Water facilities**	Unimproved water	891	3.29
	Improved	26190	96.71
**Sanitation facility**	Unimproved sanitation	9753	36.02
	Improved	17328	63.98
**Caste**	General or others	8230	30.15
	Other backward class	12762	46.75
	Scheduled castes	5038	18.46
	Scheduled tribes	1268	4.65
**Religion**	Hindu	21171	73.47
	Sikh	680	2.36
	Christian	795	2.76
	Muslims	5764	20.0
	Others	407	1.41
**Maternal covariates**	Height (cm’s) (mean)	28817	152.21
	Mother age (year) (mean)	28817	28.43
	Ever born child (mean)	28817	2.31
**Education**	Illiterate	4107	14.25
	Primary	2710	9.40
	Secondary	11543	40.06
	Higher	4185	14.52
	College	6271	21.76
**work status**	Working	841	15.88
	Not working	4455	84.12
**Breast feeding**	Yes	14764	51.23
	No	14053	48.77
**Child covariates**			
**Sex of child**	Male	15397	53.43
	Female	13420	46.57
**Child birth order**	First child	10483	36.38
	Second or third child	15155	52.59
	Four and above	3180	11.03
**Child age**	Age in months (mean)	28817	32.75
**Child had diarrhea**	No	26448	91.78
	Yes	2351	8.16
**Child had fever**	No	24797	86.05
	Yes	4010	13.92
**Child had cough**	No	25267	87.68
	Yes	3543	12.29

Note: Frequencies (n) and their respective percentage are shown for categorical variables while mean values are shown for continuous variables.

### 3.1. The prevalence of MCDBM among mother-child pairs

The distribution of different types of MCDBM among mother-child dyads is presented in Table 2. Of the 28,817 overweight/obese mothers, 6,148 had an underweight child, 7,636 had a stunted child, and 4,029 had a wasted child. The prevalence of overweight/obese mother with underweight child (OWOBM-UC), overweight/obese mother with stunted child (OWOBM-SC), and overweight/obese mother with a wasted child (i.e., OWOBM-WC) is 21.3%, 26.5%, and 14.0%, respectively. The prevalence of any form of MCDBM, overweight/obese mother–stunted or underweight or wasted child (OWOBM-SUWC) is 39% (S1 Table). The pairs of overweight/obese mother and stunted child (OWOBM-SC) is the most prevalent form MCDBM in India. In rural areas, the occurrence of OWOBM-UC (22.5%) and OWOBM-SC (29.1%) is higher while in urban areas OWOBM-WC is more prevalent. Within five wealth quintile groups, poorest households have a high prevalence of all the three forms of MCDBM. Among three forms of MCDBM, OWOBM-SC is the highest (45%) prevalent form in the poorest wealth quintile households. Exposure to poor water, sanitation, and hygiene (WASH) practices has detrimental effects on health and nutritional outcomes [50]. The prevalence of OWOBM-SC is 24.1% and 34% in the households not having access to improved water and sanitation facilities. Among social classes, Scheduled tribes (STs) and Scheduled castes (SCs) households have high occurrence of MCDBM. Muslim households suffer more from MCDBM compared to other religious groups and they have 30.3% and 23.3% OWOBM-SC and OWOBM-UC prevalence.

**Table 2:** Distribution of dual burden of malnutrition (overweight or obese mother and undernourished child) by familial, maternal and child covariates.

Household covariates		Overweight/obese mother—underweight child	Overweight/obese mother—stunted child	Overweight/obese mother–wasted child	Overweight/ obese Mothers, BMI>25
		Weighted-n (%)	Weighted-n (%)	Weighted-n (%)	Weighted number
**Residence**	Urban	2863 (20.2)	3382 (23.8)	2024 (14.3)	14183
	Rural	3285 (22.5)	4254 (29.1)	2005 (13.7)	14634
**Wealth quantile**	Poorest	618 (33.9)	820 (45.0)	278 (15.3)	1822
	Poorer	960 (28.1)	1200 (35.1)	571 (16.7)	3417
	Middle	1444 (25.5)	1888 (33.3)	755 (13.3)	5673
	Richer	1542 (20.1)	1979 (24.2)	1094 (13.4)	8171
	Richest	1483 (15.2)	1748 (18.0)	1328 (13.7)	9732
**Water facilities**	Unimproved water	206 (23.1)	215 (24.1)	125 (14.1)	891
	Improved	5635 (21.5)	7041 (26.9)	3684 (14.1)	26190
**Sanitation facility**	Unimproved sanitation	2651 (27.2)	3320 (34.0)	1460 (15.0)	9753
	Improved	3189 (18.4)	3936 (22.7)	2349 (13.6)	17328
**Caste**	General or others	1424 (17.3)	1786 (21.7)	1053 (12.8)	8230
	Other backward class	2838 (22.2)	3454 (27.1)	1909 (15.0)	12672
	Scheduled castes	1300 (25.8)	1623 (32.2)	656 (13.0)	5038
	Scheduled tribes	328 (25.9)	389 (30.7)	229 (18.1)	1268
**Religion**	Hindu	4465 (21.1)	5472 (25.8)	2981 (14,1)	21171
	Sikh	95 (14.0)	122 (17.9)	96 (14.1)	680
	Christian	140 (17.6)	163 (20.5)	98 (12.4)	795
	Muslims	1345 (23.3)	1744 (30.3)	790 (13.7)	5764
	Others	104 (25.6)	136 (33.4)	63 (15.4)	407
**Maternal covariates**					
**Height**	Above 160 cm	303 (11.6)	381 (14.9)	316 (12.1)	2619
	155 to 160 cm	983 (15.1)	1189 (18.2)	864 (13.3)	6520
	150 to 154 cm	2033 (19.8)	2642 (25.7)	1412 (13.7)	10282
	145 to 149 cm	1920 (28.9)	2271 (34.2)	1006 (15.1)	6646
	Below 145 cm	910 (33.1)	11142 (41.5)	430 (15.6)	2751
**Mothers age**	15 to 25 yeas	1870 (21.5)	2501 (28.7)	1303 (15.0)	8710
	26 to 35 years	3618 (20.9)	4398 (25.0)	2334 (13.4)	17567
	36 to 49 years	601 (23.7)	738 (29.0)	391 (15.4)	2540
**Education**	Illiterate	1279 (31.2)	1670 (40.7)	632 (15.4)	4107
	Primary	715 (26.4)	959 (35.4)	373 (13.8)	2710
	Secondary	2609 (22.6)	3109 (26.9)	1629 (14.1)	11543
	Higher	670 (16.0)	866 (20.7)	575 (13.7)	4185
	College	874 (13.9)	1032 (16.5)	819 (13.1)	6271
**Children ever born**	Single child	1218 (16.6)	2234 (21.3)	1077 (14.6)	7362
	2 or 3 children	3820 (21.6)	4153 (27.4)	2449 (13.8)	17726
	4 and more children	1110 (29.8)	1250 (39.3)	502 (13.5)	3729
**Breast feeding**	Yes	3287 (22.3)	4063 (27.5)	2286 (15.5)	14764
	No	2862 (20.4)	3573 (25.4)	1743 (12.4)	14053
**Work status**	Working	187 (19.1)	232 (27.6)	103 (12.3)	841
	Not working	851 (22.2)	1110 (24.9)	693 (15.6)	4455
**Child covariates**					
**Sex of child**	Male	3348 (21.7)	4186 (27.2)	2245 (14.6)	15397
	Female	2801 (20.9)	3451 (25.7)	1784 (13.3)	13429
**Child birth order**	First child	1880 (17.9)	2234 (21.3)	1432 (13.7)	10483
	Second or third child	3312 (21.9)	4153 (27.4)	2153 (14.2)	15155
	Four and above	956 (30.1)	1250(39.3)	444 (13.9)	3180
**Age in months**	Less than 13 months	796 (16.6)	742 (15.4)	1170 (24.3)	4807
	13 to 24 months	873 (18.4)	1516 (31.9)	582(12.2)	4752
	25 to 59 months	4479 (23.3)	5378 (27.9)	2277 (11.8)	19228
**Child had diarrhea**	No	5619 (21.2)	6993 (26.4)	3694 (14.0)	26448
	Yes	528 (22.4)	634 (27.0)	333 (14.2)	2351
**Child had fever**	No	5305 (21.4)	6594 (26.6)	5496 (14.1)	24797
	Yes	842 (21.0)	1037 (25.9)	531 (13.2)	4010
**Child had cough**	No	5460 (21.6)	6749 (26.7)	3632 (14.4)	25267
	Yes	686 (19.4)	884 (25.0)	395 (11.2)	3543

The prevalence of OWOBM-UC and OWOBM-SC among illiterate and short stature mothers is more than twice, compared to a college graduate and a mother with a height of 160 cm or more. The occurrence MCDBM is higher among overweight/obese mother and male child-pairs (27.2%) compared to a female child-paired (25.7%) with an overweight/obese mother (Table 2). If the age of the child is less than one year, the major form of MCDBM is OWOBM-WC (24.3%), but as the age of child increase to two-years, high occurrence shift to OWOBM-SC (31.9%). The prevalence of double-burden does not change significantly, whether a child has had cough, fever, or diarrhoea in the last two weeks from the date of the survey or not [47].

### 3.2. Factors associated with double burden of maternal and child malnutrition

As the dependent variables in our models are categorical and binary in nature, the most appropriate method for the analysis is the logistic regression. Table 3 depicts the results of bivariate and multivariable logistic regression for overweight/obese mother–underweight child (OWOBM-UC) pairs. Several households, maternal, and child related factors are significantly associated with MCDBM in India. Rural residency compared to urban has less likelihood of occurrence of OWOBM-UC (Adjusted aOR = 0.83; 95% CI [0.74–0.93]). Other statistically significant predictors of OWOBM-UC include unimproved sanitation facility (uOR = 1.66; 95% CI [1.50–1.82]), other-backward classes (aOR = 1.22; 95% CI [1.07–1.39]), Scheduled Castes (aOR = 1.30; 95% CI [1.10–1.53]), mother’s short height (aOR = 2.70; 95% CI [2.08–3.52]), mother’s primary education (aOR = 1.31; 95% CI [1.04–1.65]), and illiteracy (aOR = 1.48; 95% CI [1.19–1.84]), currently not breastfeeding mothers (aOR = 0.77; 95% CI [0.68–0.87]), child birth order (aOR = 1.23; 95% CI [1.04–1.46]) for second or third child and (aOR = 1.41; 95% CI [1.02–1.95]) for fourth and higher birth order, child aged 13–59 months (aOR = 1.28; 95% CI [1.08–1.52]) for 13–25 months, (aOR = 1.77; 95% CI [1.51–2.08]) for 25–59 moths and those from middle, poorer and poorest wealth quintiles (aOR = 1.40 9% CI [1.17–1.68]), (aOR = 1.46; 95% CI [1.18–1.81]) and (aOR = 1.68; 95% CI [1.32–2.14]), respectively.

**Table 3:** Logistic regression results of overweight or obese mother and underweight child by familial, maternal, and child covariates.

Household covariates		Overweight or obese mother and underweight child pairs			
		uORs [CI]^1^	*p-value*	aORs^2^ [CI]	*p-value*
**Residence**	Urban^®^	Reference (1.0)	-		
	Rural	1.15, [1.04–1.26]	0.007	0.83, [0.74–0.93]	0.001
**Wealth quintile**	Poorest	2.86, [2.43–3.36]	<0.001	1.68, [1.32–2.14]	<0.001
	Poorer	2.17, [1.86–2.53]	<0.001	1.46, [1.18–1.81]	0.001
	Middle	1.90, [1.64–2.20]	<0.001	1.40, [1.17–1.67]	<0.001
	Richer	1.40, [1.21–1.62]	<0.001	1.18, [1.01–1.38]	0.040
	Richest^®^	Reference (1.0)	-		
**Water facilities**	Unimproved water	1.09, [0.87–1.38]	0.447		
	Improved^®^	Reference (1.0)	-		
**Sanitation facility**	Unimproved sanitation	1.66, [1.50–1.82]	<0.001	1.08, [0.96–1.22]	0.212
	Improved ^®^	Reference (1.0)			
**Caste**	General or others^®^	Reference (1.0)	-		
	Other backward class	1.37, [1.21–1.54]	<0.001	1.22, [1.07–1.39]	0.003
	Scheduled castes	1.66, [1.43–1.93]	<0.001	1.30, [1.10–1.53]	0.002
	Scheduled tribes	1.67, [1.37–2.04]	<0.001	1.23, [0.98–1.55]	0.071
**Religion**	Hindu^®^	Reference (1.0)	-		
	Sikh	0.61, [0.47–0.78]	<0.001	0.86, [0.66–1.12]	0.274
	Christian	0.80, [0.60–1.064]	0.124	0.95, [0.69–1.29]	0.730
	Muslims	1.14, [1.01–1.29]	0.036	1.12, [0.97–1.28]	0.123
	Others	1.29, [0.86–1.93]	0.220	1.21, [0.77–1.90]	0.409
**Maternal covariates**					
**Height**	Above 160 cm^®^	Reference (1.0)	-		
	155 to 160 cm	1.36, [1.07–1.72]	0.011	1.26, [0.98–1.61]	0.071
	150 to 154 cm	1.88, [1.52–2.34]	<0.001	1.58, [1.26–1.99]	<0.001
	145 to 149 cm	3.10, [2.46–3.91]	<0.001	2.43, [1.90–3.10]	<0.001
	Below 145 cm	3.78, [2.94–4.85]	<0.001	2.70, [2.08–3.52]	<0.001
**Age**	26 to 35 years^®^	Reference (1.0)	-		
	15 to 25 yeas	1.03, [0.93–1.15]	0.556	1.11, [0.98–1.26]	0.101
	36 to 49 years	1.17, [1.01–1.37]	0.043	1.00, [0.84–1.18]	0.963
**Education**	Illiterate	2.79, [2.38–3.27]	<0.001	1.48, [1.19–1.84]	<0.001
	Primary	2.21, [1.83–2.67]	<0.0001	1.31, [1.04–1.65]	0.020
	Secondary	1.80, [1.55–2.10]	<0.0001	1.31, [1.09–1.58]	0.004
	Higher	1.18, [0.98–1.42]	0.087	0.99, [0.81–1.22]	0.934
	College^®^	Reference (1.0)			
**Children ever born**	Single child^®^	Reference (1.0)	-		
	2 or 3 children	1.39, [1.22–1.57]	<0.001	1.02, [0.84–1.24]	0.823
	4 and more children	2.14, [1.85–2.47]	<0.001	1.00, [0.71–1.40]	0.998
**Breast feeding**	Yes^®^	Reference (1.0)	-		
	No	0.90, [0.81–0.98]	0.022	0.77, [0.68–0.87]	<0.001
**work status**	Working^®^	Reference (1.0)	-		
	Not working	0.83, [0.64–1.07]	0.153		
**Child covariates**					
**Sex of child**	Male^®^	1.05, [0.96–1.16]	0.279		
	Female	Reference (1.0)			
**Child birth order**	First child^®^	Reference (1.0)	-		
	Second or third child	1.28, [1.15–1.42]	<0.001	1.23, [1.04–1.46]	0.016
	Four and above	1.97, [1.73–2.24]	<0.001	1.41, [1.02–1.95]	0.038
**Age in months**	Less than 13 months^®^	Reference (1.0)	-		
	13 to 24 months	1.13, [0.97–1.32]	0.109	1.28, [1.08–1.52]	0.004
	25 to 59 months	1.53, [1.35–1.73]	<0.001	1.77, [1.51–2.08]	<0.001
**Child had diarrhea**	No^®^	Reference (1.0)	-		
	Yes	1.07, [0.93–1.24]	0.356		
**Child had fever**	No ^®^	Reference (1.0)	-		
	Yes	0.98, [0.86–1.11]	0.712		
**Child had cough**	(No) ^®^	Reference (1.0)	-		
	Yes	0.87, [0.77–0.99]	0.034	0.87, [0.76–1.00]	0.052

^1^.Odds ratios and confidence interval at 5% significance level of bivariate regression models.

^2^. Odds ratios in multivariable model after adjusting for the variables which were significant at 5% level in bivariate model.

There are other determinants which are significant in bivariate model. These are mothers aged 36–49 years compared to 25–35 years (uOR = 1.17; 95% CI [1.01–1.37]), mother given birth to four or more children, and Muslim households (uOR = 2.14; 95% CI [1.85–2.47]), (uOR = 1.14 95% CI [1.01–1.29]), respectively. Other factors (sex of child, child’s health, mother’s work status, and improved/unimproved water facility) are not significantly associated with the likelihood of OWOBM-UC.

Table 4 depicts the logistic regression results for overweight/obese mother- stunted child (OWOBM-SC) dyads. In bivariate model the likelihood of occurrence of OWOBM-SC is higher in rural areas (Unadjusted uOR = 1.31; 95% CI [1.20–1.44]) but in multivariable model the likelihood of OWOBM-SC is less in rural area and it is statistically insignificant. Other predictors, which are significant in occurrence of OWOBM-SC are following: other-backward class (aOR = 1.17; 95% CI [1.04–1.31]), Scheduled Castes (aOR = 1.31; 95% CI [1.13–1.52]), mother’s short stature (aOR = 2.94; 95% CI [2.30–3.75]), maternal illiteracy (aOR = 1.58; 95% CI [1.29–1.93]), and primary education (aOR = 1.44; 95% CI [1.16–1.79]). Households with mothers in age group 15–25 years has higher probability (aOR = 1.36; 95% CI [1.21–1.53]) of MCDBM in comparison to 26–36 years aged mothers. Number of children ever-born are significant (uOR = 2.50; 95% CI [1.19–2.85]) in bivariate model, but when we control the other variables which are significant in bivariate model then it became insignificant.

**Table 4:** Logistic regression results of overweight /obese mother and stunted child by familial, maternal, and child covariates.

Household covariate		Overweight or obese mother and stunted child pairs			
		uORs [CI]^1^	*p-value*	aORs^2^ [CI]	*p-value*
**Residence**	Urban^®^	Reference (1.0)	-		
	Rural	1.31, [1.20–1.44]	<0.001	0.91, [0.82–1.01]	0.083
**Wealth quintile**	Poorest	3.74, [3.17–4.41]	<0.001	2.01, [1.59–2.54]	<0.001
	Poorer	2.47, [2.14–2.85]	<0.001	1.49, [1.23–1.80]	<0.001
	Middle	2.28, [1.99–2.61]	<0.001	1.63, [1.38–1.93]	<0.001
	Richer	1.45, [1.28–1.66]	<0.001	1.16, [1.01–1.34]	0.034
	Richest^®^	Reference (1.0)	-		
**Water facilities**	Unimproved water	0.87, [0.70–1.08]	0.190		
	Improved^®^	Reference (1.0)	-		
**Sanitation facility**	Unimproved sanitation	1.76, [1.61–1.92]	<0.001	1.05, [0.94–1.17]	0.391
	Improved ^®^	Reference (1.0)			
**Caste**	General or others^®^	Reference (1.0)	-		
	Other backward class	1.34, [1.20–1.49]	<0.001	1.17, [1.04–1.31]	0.010
	Scheduled castes	1.71, [1.50–1.96]	<0.001	1.31, [1.13–1.52]	<0.001
	Scheduled tribes	1.60, [1.32–1.94]	<0.001	1.05, [0.83–1.32]	0.689
**Religion**	Hindu^®^	Reference (1.0)	-		
	Sikh	.62, [0.49–0.80]	<0.001	0.90, [0.70–1.17]	0.437
	Christian	.74, [0.57–0.95]	0.019	0.91, [0.70–1.18]	0.464
	Muslims	1.24, [1.12–1.37]	<0.001	1.11, [0.98–1.26]	0.099
	Others	1.44, [0.91–2.28]	0.120	1.34, [0.81–2.22]	0.251
**Maternal covariates**					
**Height**	Above 160 cm^®^	Reference (1.0)	-		
	155 to 160 cm	1.27, [1.04–1.55]	0.018	1.14, [0.93–1.40]	0.223
	150 to 154 cm	1.97, [1.63–2.38]	<0.001	1.68, [1.37–2.05]	<0.001
	145 to 149 cm	2.96, [2.43–3.60]	<0.001	2.38, [1.92–2.94]	<0.001
	Below 145 cm	4.05, [3.23–5.07]	<0.001	2.94, [2.30–3.75]	<0.001
**Mothers age**	26 to 35 years^®^	Reference (1.0)	-		
	15 to 25 yeas	1.21, [1.09–1.33]	<0.001	1.36, [1.21–1.53]	<0.001
	36 to 49 years	1.23, [1.06–1.41]	<0.001	1.00, [0.86–1.17]	0.979
**Education**	Illiterate	3.48, [2.99–4.04]	<0.001	1.58, [1.29–1.93]	<0.001
	Primary	2.78, [2.33–3.32]	<0.0001	1.44, [1.16–1.79]	0.001
	Secondary	1.87, [1.62–2.16]	<0.0001	1.25, [1.06–1.47]	0.009
	Higher	1.32, [1.10–1.59]	0.002	1.11, [0.91–1.34]	0.300
	College^®^	Reference (1.0)	-		
**Children ever born**	Single child^®^	Reference (1.0)	-		
	2 or 3 children	1.44, [1.28–1.62]	<0.001	0.98, [0.81–1.17]	0.780
	4 and more children	2.50, [2.19–2.85]	<0.001	0.95, [0.70–1.30]	0.758
**Breast feeding**	Yes^®^	Reference (1.0)	-		
	No	0.90	0.024	0.80, [0.71–0.91]	<0.001
**work status**	Working^®^	Reference (1.0)	-		
	Not working	0.87, [0.693–1.10]	0.240		
**Child covariates**					
**Sex of child**	Male^®^	1.08, [0.99–1.18]	0.096		
	Female	Reference (1.0)			
**Child birth order**	First child^®^	Reference (1.0)	-		
	Second or third child	1.40, [1.26–1.54]	<0.001	1.49, [1.27–1.74]	<0.001
	Four and above	2.40, [2.12–2.70]	<0.001	1.90, [1.40–2.58]	<0.001
**Age in months**	Less than 13 months^®^	Reference (1.0)	-		
	13 to 24 months	2.57, [2.20–2.99]	<0.001	3.29, [2.76–3.92]	<0.001
	25 to 59 months	2.12, [1.87–2.41]	<0.001	2.69, [2.29–3.17]	<0.001
**Child had diarrhoea**	No^®^	Reference (1.0)	-		
	Yes	1.03, [0.90–1.17]	0.682		
**Child had fever**	No ^®^	Reference (1.0)	-		
	Yes	0.96, [0.86–1.08]	0.514		
**Child had cough**	(No) ^®^	Reference (1.0)	-		
	Yes	0.91, [0.81–1.02]	0.119		

^1^. Odds ratios and confidence interval at 5% significance level of bivariate regression models.

^2^. Odds ratios in multivariable model after adjusting for the variables which were significant at 5% level in bivariate models.

Child aged 13–59 months and wealth index are also significant predictors of MCDBM of OWOBM-SC. Odds ratio in favour of occurrence of OWOBM-SC is (aOR = 3.29; 95% CI [2.76–3.92]) for 13–25 months and (aOR = 2.69; 95% CI [2.29–3.17]) for 25–59 months. The likelihood of OWOBM-SC dyads among middle, poorer, and poorest wealth quintiles is (aOR = 1.63 9% CI [1.38–1.93]), (aOR = 1.49; 95% CI [1.23–1.80]), and (aOR = 2.01; 95% CI [1.59–2.54]), respectively; indicating higher burden of maternal and child malnutrition among the poor.

Table 5 shows the results for the overweight/obese mother with wasted child (OWOBM-WC) pairs. The pair of mother-child in the households without improved sanitation facility is more likely (uOR = 1.12; 95% CI [0.99–1.26]) to be an OWOBM-WC pair compared to households with improved sanitation facilities. Being in poorer household also increases the likelihood of occurrence of OWOBM-WC with an odds ratio (aOR = 1.23; 95% CI [1.02–1.48]). Caste is another significant predictor in both bivariate and multivariable models (aOR = 1.45; 95% CI [1.13–1.87]) for STs and (aOR = 1.20; 95% CI [1.05–1.38]) for other-backward class.

**Table 5:** Logistic regression results of overweight / obese mother and wasted child by familial, maternal and child covariates.

Household covariates		Overweight or obese mother and wasted child pairs			
		uORs [CI]^1^	*p-value*	aORs^2^ [CI]	*p-value*
**Residence**	Urban^®^	Reference (1.0)	-		
	Rural	0.95, [0.85–1.07]	0.419		
**Wealth quintile**	Poorest	1.15, [0.94–1.40]	0.183	1.06, [0.86–1.31]	0.595
	Poorer	1.27, [1.06–1.52]	0.009	1.23, [1.02–1.48]	0.033
	Middle	0.97, [0.83–1.14]	0.731	0.92, [0.78–1.09]	0.333
	Richer	0.98, [0.84–1.14]	0.780	0.95, [0.82–1.12]	0.547
	Richest^®^	Reference (1.0)	-		
**Water facilities**	Unimproved water	1.00, [0.78–1.28]	0.998		
	Improved^®^	Reference (1.0)	-		
**Sanitation facility**	Unimproved sanitation	1.12, [1.00–1.26]	0.051		
	Improved ^®^	Reference (1.0)	-		
**Caste**	General or others^®^	Reference (1.0)	-		
	Other backward class	1.20, [1.05–1.37]	0.009	1.20, [1.05–1.38]	0.008
	Scheduled castes	1.02, [0.86–1.21]	0.820	0.99, [0.83–1.18]	0.923
	Scheduled tribes	1.50, [1.17–1.93]	0.001	1.45, [1.13–1.87]	0.004
**Religion**	Hindu^®^	Reference (1.0)	-		
	Sikh	1.00, [0.77–1.30]	0.999		
	Christian	0.86, [0.62–1.20]	0.385		
	Muslims	0.97, [0.84–1.11]	0.664		
	Others	1.11, [0.66–1.88]	0.693		
**Maternal covariates**					
**Height**	Above 160 cm^®^	Reference (1.0)	-		
	155 to 160 cm	1.11, [0.89–1.39]	0.355	1.12, [0.89–1.41]	0.346
	150 to 154 cm	1.16, [0.94–1.42]	0.161	1.19, [0.96–1.47]	0.118
	145 to 149 cm	1.30, [1.05–1.61]	0.016	1.33, [1.07–1.66]	0.011
	Below 145 cm	1.35, [1.05–1.72]	0.018	1.38, [1.07–1.78]	0.012
**Mothers age**	26 to 35 years^®^	Reference (1.0)	-		
	15 to 25 yeas	1.15, [1.02–1.29]	0.019	1.01, [0.90–1.14]	0.830
	36 to 49 years	1.19, [0.97–1.46]	0.102	1.19, [0.96–1.48]	0.111
**Education**	Illiterate	1.21, [1.10–1.44]	0.029		
	Primary	1.06, [0.87–1.30]	0.557		
	Secondary	1.09, [0.93–1.28]	0.270		
	Higher	1.06, [0.89–1.27]	0.520		
	College^®^	Reference (1.0)	-		
**Children ever born**	Single child^®^	Reference (1.0)	-		
	2 or 3 children	0.94, [0.83–1.06]	0.290		
	4 and more children	0.91, [0.77–1.07]	0.271		
**Breast feeding**	Yes^®^	Reference (1.0)	-		
	No	0.77, [0.69–0.87]	<0.001	1.06, [0.94–1.20]	0.324
**work status**	Working^®^	Reference (1.0)	-		
	Not working	1.32, [0.98–1.78]	0.070		
**Child covariates**					
**Sex of child**	Male^®^	1.11, [0.99–1.25]	0.063		
	Female	Reference (1.0)	-		
**Child birth order**	First child^®^	Reference (1.0)	-		
	Second or third child	1.05, [0.94–1.17]	0.419		
	Four and above	1.03, [1.02–1.29]	0.779		
**Age in months**	Less than 13 months^®^	Reference (1.0)	-		
	13 to 24 months	0.43, [0.37–0.51]	<0.001	0.41, [0.35–0.49]	<0.001
	25 to 59 months	0.42, [0.37–0.47]	<0.001	0.39, [0.34–0.45]	<0.001
**Child had diarrhoea**	No^®^	Reference (1.0)	-		
	Yes	1.02, [0.84–1.23]	0.867		
**Child had fever**	No ^®^	Reference (1.0)	-		
	Yes	0.93, [0.79–1.10]	0.393		
**Child had cough**	(No) ^®^	Reference (1.0)	-		
	Yes	0.75, [0.64–0.88]	0.001	0.73, [0.61–0.86]	<0.001

^1^.Odds ratios and confidence interval at 5% significance level of bivariate regression models

^2^. Odds ratios in multivariable model after adjusting for the variables which were significant at 5% level in bivariate models.

Maternal height is not a significant predictor of MCDBM of OWOBM-WC in bivariate model but in adjusted model. Short stature of the mother is a statistically significant predictor (aOR = 1.33; 95% CI [1.07–1.66]) for height 145–149 cm and (aOR = 1.38; 95% CI [1.07–1.78]) for below 145 cm in comparison to a height of 160 cm and above. As the age of the child increases, the likelihood of occurrence of OWOBM-WC reduces significantly (aOR = 0.39, 95% CI [0.35–0.45]) for the 25–59 moths compared to below 13 months. Mothers age, education, and breastfeeding status are significant in the bivariate model but become statistically insignificant in the multivariable model after adjusting for predictors which are significant at 95% level in the bivariate model.

## 4. Discussion

The findings of the study highlight the complexities of relationship between socio-economic status and MCDBM in India. It became more important to explore these nutrition complexities as epidemiological, economic, and nutrition transition are taking place in India. Nutrition transition is reflected in changing dietary pattern and a substantial increase in overweight/obesity in recent years [34, 36, 51]. The rise in overweight/obesity happens mainly among adults, and early age children are still disproportionally undernourished. We find that stunting and underweight among the under-5 age children decreased and the prevalence is 38.3% and 35.7% in 2015–16. But unfortunately, wasting, the worst form of child malnutrition, increased between 2005–06 to 2015–16, and 21.04% children were wasted in 2015–16 compared to 19.8% in 2006. Low birth-weight and lack of awareness about complementary feeding and lower utilisation of supplementary nutrition by lactating mothers could be the possible explanations [52]. This paradoxical nature of adult (overnutrition) and child (undernutrition) malnutrition gives rise to the problem of DBM among mother-child dyads in India. The present study finds that overweight/obese mothers have a substantial percentage of undernourished children in India. These findings are consistent with other studies conducted in other LMICs.

Among LMICs countries, the prevalence of DBM was 24.3% in Bangladesh [48], (16.3%) in Indonesia [53], (9.1%) in Myanmar [54], (8%) in China [55], (6.60%) in Nepal [49], and 3(.9%) in Pakistan [47]. However, there are some methodological differences in conceptualising the MCDBM, but one thing is common that all these studies have taken mother-child pairs as a unit of analysis. The prevalence of MCDBM in a specific regions or among communities is higher than the India’s average, like in the rural area of western Java, the DBM was (30.6%) [56], 18.5% in poor areas of China [57], (15.7%) in Gaza-strip of Palestine [58], and 43% in the poor urban setting of Nairobi in Kenya [59].

The problem of MCDBM is not independent in itself rather it is driven by rapid increase in maternal BMI and a lower rate of decline in child malnutrition. We find that the most common form of double burden is in OWOBM-SC pairs and lesser prevalence in OWOBM-WC pairs. Low prevalence of child wasting (21%) compared to underweight and stunting might be the cause behind less occurrence of OBM/WC pairs [23]. The results are in accordance with other studies that reported DBM in Asia [48, 49, 53, 54]. In the literature, it is well established that co-occurrence of under- and overnutrition is associated with nutrition transition [8]. Recent studies show a rapid change in nutritional status of adults and changing food preferences [34, 36] which confirms that the nutrition transition is undergoing in India. More preference toward high-energy dense food items result in poor nutrient content for adults and child bearing mothers which in turn lead to child undernourishment [60]. This shift in dietary pattern in past decades may be a probable explanation for presence of MCDBM in India.

The study also finds that various household, maternal, and child related factors are associated differently with different form of MCDBM in India. Our results are consistent with previous studies in other LMICs countries which find less likelihood of MCDBM in rural areas and household with improved sanitation facilities [47]. Interestingly, our study contrasts with some others on association with wealth group and find higher probability of MCDBM among poorer households. This phenomenon also has an explanation that there has been a steep increase in fat calorie intake and overweight/obesity among adult population in lower income strata in India in past decades [36, 51].

Present study also attempts to identify the mother and child related factors contributing to MCDBM in India. As reported in other contemporary studies, we also find that mother’s short stature, height below 145 cm, is a strong predictor of double burden [61]. The likelihood of OWOBM-SC is the highest for maternal short stature. It is an indication of intergenerational transfer of malnutrition. It has strong policy implications because if the childhood stunting is not reduced swiftly, an increase in early-age obesity may result in a more serious individual level (stunted as well as obese) dual burden. Educated mothers have less likely to bear an undernourished child and thus lower probability of MCDBM. It may be moderated in two ways; on the one hand, educated mothers might be more aware about child feeding practices and nutritious food choices; on the other hand, more educated mothers are in rich households which has lower probability of MCDBM. Number of children ever born are significant in bivariate but after controlling the confounding factors in multivariable model, the effect is insignificant. Current working status of mother is not a significant predictor of MCDBM [47]. This may be due to the fact that non-working mothers are highly engaged in childcare activities or NFHS does not follow the standard classification for occupational activities of the mothers.

Among child related factors, age of the child and birth order are significant predictors of occurrence of OWOBM-UC and OWOBM-SC. The study has some conformity with other studies [23] and the underlying mechanism may be that after early age growth failure, there are less chances of recovery. In addition, we also find that prevalence of obesity is higher among mothers with children aged 2–5 years [49]. Sex and health of the child do not show any significant association with double burden of malnutrition [47]. In a qualitative assessment of different rounds of NFHS data, authors [62] find no significant differences for sex in prevalence of malnutrition which contrasts to other studies and raises concerns.

The present study possesses several strengths as well as some limitations. Considerable strengths are—first, the study used large scale nationally representative household survey that has high response rate and very small number of missing observations which provides the basis for reliable estimates from statistical point of view. Second, based on the premise that mother-child have closer contact with each other and share more resources than other members of the household, they should have similar nutritional status, we restrict our sample to overweight/obese mothers having at least one under-5 age child. Restricting our sample to overweight/obese mothers’ household gives an additional strength because it reveals the true picture of double burden of MCDBM in an increasingly becoming overweight/obese society. Third, in the DHS surveys, standard height and weight measuring tools are used by trained field staff to collect anthropometric measurement of the eligible respondents.

We also acknowledge the few limitations of the study. First, the study could not able to establish the causal relationship between outcome and independent variables due to the cross-sectional nature of the data. Second, the use of secondary data also poses some limitations to control some other relevant confounding factors. Third, we have used WHO body-mass index criteria for classification of overweight/obesity that may have incorporated some bias because this classification does not consider the ethnic and region specific aspects of overweight/obesity [63]. Finally, we only analyse the sociodemographic and economic determinants of DBM. Factors such as physical activity, dietary intakes, caregiving approaches, and importantly cultural aspects were not included in the database. Therefore, we expect further exploration to identify the contribution of some of these factors on development of MCDBM related to malnutrition in India.

## 5. Conclusion

The present study is an attempt to analyse whether India is facing the double burden of MCDBM. The findings of the study reveal the existence of MCDBM in India where overweight/obese mothers have significant percentage of undernourished children. Both rapid rise in adult overweight/obesity and slow progress on child undernutrition contributes to maternal and child related double burden of malnutrition. Like in other LMICs, nutrition transition is dragging upward movement in MCDBM in India. Our findings reinforce the continuation of malnutrition prevention programmes and interventions and inclusion of postpartum mothers in nutrition related awareness and behavioural counselling to prevent the growing MCDBM in India. This will help India to achieve the zero hunger and health and well-being SDGs by 2030. Strikingly, higher fat calorie intake, increasing overweight/obesity, and high prevalence of MCDBM among poor population require urgent attention of policy makers. Because such nutritional crises pose additional burden to ongoing public health policy challenge in India.

## Supporting information

S1 Table Predicted probabilities of double burden of malnutrition among mother-child dyads by familial, maternal, and child covariates in India. (DOCX)

S2 Table A sub-sample multivariable analysis with 60 percent of the total sample of double burdened mother-child pairs. (DOCX)

S3 Table Distribution of any form dual burden of malnutrition (overweight or obese mother and underweight/stunted/wasted child) by familial, maternal and child covariates. (DOCX)

S4 Table Logistic regression results of any form of dual burden of malnutrition (overweight or obese mother and underweight/stunted/wasted child) by familial, maternal and child covariates. (DOCX)

## Abbreviations

DBM: Double Burden of Malnutrition
MCDBM: Maternal and Child Double Burden of Malnutrition
OWOBM: Overweight or Obese Mother
OWOBM-SC: Overweight or Obese Mother and Stunted Child
OWOBM-SUWC: Overweight or Obese Mother and Stunted or Underweight, or Wasted Child
OWOBM-UC: Overweight or Obese Mother and Underweight Child
OWOBM-WC: Overweight or Obese Mother and Wasted Child
