# Supplementary material for: Does maternal overnutrition carry child undernutrition in India?
Source: PLoS One. 2022 Jun 17;17(6):e0265788. doi: 10.1371/journal.pone.0265788 (PMC9205528; doi:10.1371/journal.pone.0265788)
Supplement: S1 Table — (DOCX) [file pone.0265788.s001.docx]

| **S1: Predicted probabilities of double burden of malnutrition among mother-child dyads by familial, maternal, and child covariates in India.** | | | | | | | |
| --- | --- | --- | --- | --- | --- | --- | --- |
| **Characteristics**  **Household covariates** | | **Overweight or obese mother and underweight child pairs** | | **Overweight or obese mother and stunted child pairs** | | **Overweight or obese mother and wasted child pairs** | |
|  |  | **Predicted probability^1^ , [ CI]^2^** | ***p-value*** | **Predicted probability , [CI]** | ***p-value*** | **Predicted probability , [CI]** | ***p-value*** |
| **Residence** | Urban^®^ | 0.24 [0.22-0.25] | <0.001 | 0.28 [0.26 - 0.29] | <0.001 |  |  |
|  | Rural | 0.20 [0.19-0.21] | <0.001 | 0.26 [0.25 - 0.27] | <0.001 |  |  |
| **Wealth quantile** | Poorest | 0.27 [0.24-0.31] | <0.001 | 0.36 [0.32 - 0.39] | <0.001 | 0.15 [0.13 - 0.17] | <0.001 |
|  | Poorer | 0.25 [0.22-0.27] | <0.001 | 0.29 [0.27 - 0.32] | <0.001 | 0.17 [0.15 - 0.19] | <0.001 |
|  | Middle | 0.24 [0.22-0.26] | <0.001 | 0.31 [0.29 - 0.33] | <0.001 | 0.13 [0.12 - 0.14] | <0.001 |
|  | Richer | 0.21 [0.19-0.23] | <0.001 | 0.25 [0.23 - 0.26] | <0.001 | 0.14 [0.12 - 0.15] | <0.001 |
|  | Richest^®^ | 0.19 [0.17-0.2] | <0.001 | 0.22 [0.2 - 0.24] | <0.001 | 0.14 [0.13 - 0.15] | <0.001 |
| **Sanitation facility** | Unimproved sanitation | 0.23 [0.21-0.24] | <0.001 | 0.27 [0.26 - 0.29] | <0.001 |  |  |
|  | Improved ^®^ | 0.21 [0.20-0.22] | <0.001 | 0.27 [0.25 - 0.28] | <0.001 |  |  |
| **Caste** | General or others^®^ | 0.19 [0.18-0.21] | <0.001 | 0.25 [0.23 - 0.26] | <0.001 | 0.13 [0.12 - 0.14] | <0.001 |
|  | Other backward class | 0.22 [0.21-0.24] | <0.001 | 0.27 [0.26 - 0.28] | <0.001 | 0.15 [0.14 - 0.16] | <0.001 |
|  | Scheduled castes | 0.24 [0.22-0.25] | <0.001 | 0.29 [0.27 - 0.32] | <0.001 | 0.13 [0.11 - 0.14] | <0.001 |
|  | Scheduled tribes | 0.23 [0.19-0.26] | <0.001 | 0.25 [0.22 - 0.29] | <0.001 | 0.18 [0.14 - 0.21] | <0.001 |
| **Religion** | Hindu^®^ | 0.22 [0.21-0.22] | <0.001 | 0.27 [0.26 - 0.28] | <0.001 |  |  |
|  | Sikh | 0.19 [0.15-0.23] | <0.001 | 0.25 [0.2 - 0.29] | <0.001 |  |  |
|  | Christian | 0.21 [0.16-0.25] | <0.001 | 0.25 [0.21 - 0.29] | <0.001 |  |  |
|  | Muslims | 0.23 [0.21-0.25] | <0.001 | 0.28 [0.26 - 0.3] | <0.001 |  |  |
|  | Others | 0.25 [0.17-0.33] | <0.001 | 0.32 [0.22 - 0.42] | <0.001 |  |  |
| **Maternal covariates** |  |  |  |  |  |  |  |
| **Height** | Above 160 cm^®^ | 0.14 [0.11-0.17] | <0.001 | 0.18 [0.15 - 0.2] | <0.001 | 0.12 [0.1 - 0.14] | <0.001 |
|  | 155 to 160 cm | 0.17 [0.15-0.19] | <0.001 | 0.2 [0.18 - 0.21] | <0.001 | 0.13 [0.12 - 0.15] | <0.001 |
|  | 150 to 154 cm | 0.2 [0.19-0.22] | <0.001 | 0.26 [0.25 - 0.28] | <0.001 | 0.14 [0.13 - 0.15] | <0.001 |
|  | 145 to 149 cm | 0.28 [0.26-0.3] | <0.001 | 0.33 [0.31 - 0.35] | <0.001 | 0.15 [0.14 - 0.17] | <0.001 |
|  | Below 145 cm | 0.3 [0.27-0.33] | <0.001 | 0.37 [0.34 - 0.41] | <0.001 | 0.16 [0.14 - 0.18] | <0.001 |
| **Mothers age** | 26 to 35 years^®^ | 0.23 [0.21-0.25] | <0.001 | 0.31 [0.29 - 0.33] | <0.001 | 0.14 [0.13 - 0.15] | <0.001 |
|  | 15 to 25 yeas | 0.21 [0.2-0.22] | <0.001 | 0.25 [0.24 - 0.26] | <0.001 | 0.14 [0.13 - 0.15] | <0.001 |
|  | 36 to 49 years | 0.21 [0.19-0.24] | <0.001 | 0.25 [0.23 - 0.28] | <0.001 | 0.16 [0.13 - 0.19] | <0.001 |
| **Education** | Illiterate | 0.25 [0.23-0.27] | <0.001 | 0.31 [0.29 - 0.34] | <0.001 |  |  |
|  | Primary | 0.23 [0.2-0.25] | <0.001 | 0.30 [0.27 - 0.32] | <0.001 |  |  |
|  | Secondary | 0.23 [0.21-0.24] | <0.001 | 0.27 [0.25 - 0.28] | <0.001 |  |  |
|  | Higher | 0.18 [0.16-0.2] | <0.001 | 0.25 [0.22 - 0.27] | <0.001 |  |  |
|  | College^®^ | 0.19 [0.16-0.21] | <0.001 | 0.23 [0.21 - 0.25] | <0.001 |  |  |
| **Children ever born** | Single child^®^ | 0.22 [0.19-0.24] | <0.001 | 0.27 [0.24 - 0.3] | <0.001 |  |  |
|  | 2 or 3 children | 0.22 [0.21-0.23] | <0.001 | 0.27 [0.26 - 0.28] | <0.001 |  |  |
|  | 4 and more children | 0.22 [0.18-0.25] | <0.001 | 0.26 [0.23 - 0.3] | <0.001 |  |  |
| **Breast feeding** | Yes^®^ | 0.20 [0.19-0.21] | <0.001 | 0.25 [0.24 - 0.26] | <0.001 | 0.15 [0.13 - 0.16] | <0.001 |
|  | No | 0.24 [0.23-0.25] | <0.001 | 0.29 [0.27 - 0.3] | <0.001 | 0.14 [0.13 - 0.15] | <0.001 |
| **Child covariates** |  |  |  |  |  |  |  |
| **Child birth order** | First child^®^ | 0.19 [0.17-0.21] | <0.001 | 0.22 [0.2 - 0.24] | <0.001 |  |  |
|  | Second or third child | 0.23 [0.21-0.24] | <0.001 | 0.29 [0.27 - 0.3] | <0.001 |  |  |
|  | Four and above | 0.25 [0.21-0.29] | <0.001 | 0.34 [0.29 - 0.38] | <0.001 |  |  |
| **Age in months** | Less than 13 months^®^ | 0.16 [0.14-0.17] | <0.001 | 0.14 [0.12 - 0.15] | <0.001 | 0.25 [0.23 - 0.28] | <0.001 |
|  | 13 to 24 months | 0.19 [0.17-0.21] | <0.001 | 0.33 [0.3 - 0.35] | <0.001 | 0.12 [0.11 - 0.14] | <0.001 |
|  | 25 to 59 months | 0.24 [0.23-0.25] | <0.001 | 0.29 [0.28 - 0.3] | <0.001 | 0.12 [0.11 - 0.13] | <0.001 |
| **Cough** | (No) ^®^ | 0.22 [0.21-0.23] | <0.001 |  |  | 0.15 [0.14 - 0.15] | <0.001 |
|  | Yes | 0.20 [0.18-0.22] | <0.001 |  |  | 0.11 [0.1 - 0.13] | <0.001 |

1. Predicted probability of the variables which were included in multivariable logistic regression models.

2. Confidence interval at 95 % significance level.
