# Supplementary material for: Does maternal overnutrition carry child undernutrition in India?
Source: PLoS One. 2022 Jun 17;17(6):e0265788. doi: 10.1371/journal.pone.0265788 (PMC9205528; doi:10.1371/journal.pone.0265788)
Supplement: S2 Table — (DOCX) [file pone.0265788.s002.docx]

| **S2: A sub-sample multivariable analysis with 60 percent of the total sample of double burdened mother-child pairs.** | | | | | | | |
| --- | --- | --- | --- | --- | --- | --- | --- |
| **Characteristics**  **Household covariates** | | **Overweight or obese mother and underweight child pairs** | | **Overweight or obese mother and stunted child pairs** | | **Overweight or obese mother and wasted child pairs** | |
|  |  | **Odds Ratio, [95 %, CI]^1^** | ***p-value*** | **Odds Ratio, [95 %, CI]** | ***p-value*** | **Odds Ratio, [95 % , CI]** | ***p-value*** |
| **Residence** | Urban^®^ | Ref. (1.0) | - | Ref. (1.0) | - | Ref. (1.0) | - |
|  | Rural | 0.86, [0.74- 0.99] | 0.041 | 0.95, [0.83- 1.09] | 0.443 | - | - |
| **Wealth quantile** | Poorest | 1.74, [1.28- 2.37] | < 0.001 | 2.17, [1.6- 2.96] | < 0.001 | 1.06, [0.8- 1.41] | 0.665 |
|  | Poorer | 1.3, [0.99- 1.72] | 0.058 | 1.38, [1.08- 1.77] | 0.010 | 1.21, [0.95- 1.54] | 0.130 |
|  | Middle | 1.36, [1.09- 1.71] | 0.007 | 1.54, [1.25- 1.89] | < 0.001 | 0.93, [0.75- 1.15] | 0.505 |
|  | Richer | 1.1, [0.9- 1.36] | 0.342 | 1.05, [0.87- 1.27] | 0.594 | 0.98, [0.81- 1.18] | 0.823 |
|  | Richest^®^ | Ref. (1.0) | - | Ref. (1.0) | - | Ref. (1.0) | - |
| **Sanitation facility** | Unimproved sanitation | 1.03, [0.89- 1.21] | 0.680 | 0.99, [0.86- 1.14] | 0.869 | - | - |
|  | Improved ^®^ | Ref. (1.0) | - | Ref. (1.0) | - | Ref. (1.0) | - |
| **Caste** | General or others^®^ | Ref. (1.0) | - | Ref. (1.0) | - | Ref. (1.0) | - |
|  | Other backward class | 1.24, [1.05- 1.46] | 0.009 | 1.2, [1.03- 1.41] | 0.020 | 1.21, [1.01- 1.46] | 0.042 |
|  | Scheduled castes | 1.41, [1.14- 1.75] | 0.001 | 1.4, [1.15- 1.71] | 0.001 | 1.02, [0.81- 1.29] | 0.861 |
|  | Scheduled tribes | 1.14, [0.85- 1.54] | 0.381 | 1.06, [0.81- 1.39] | 0.667 | 1.51, [1.11- 2.05] | 0.010 |
| **Religion** | Hindu^®^ | Ref. (1.0) | - | Ref. (1.0) | - | Ref. (1.0) | - |
|  | Sikh | 0.88, [0.64- 1.2] | 0.415 | 1.01, [0.75- 1.34] | 0.969 | - | - |
|  | Christian | 1.01, [0.66- 1.54] | 0.974 | 0.95, [0.67- 1.34] | 0.776 | - | - |
|  | Muslims | 1.2, [1.01- 1.43] | 0.035 | 1.11, [0.95- 1.29] | 0.175 | - | - |
|  | Others | 1.23, [0.7- 2.14] | 0.475 | 1.29, [0.76- 2.21] | 0.343 | - | - |
| **Maternal covariates** |  |  |  |  |  |  |  |
| **Height** | Above 160 cm^®^ | Ref. (1.0) | - | Ref. (1.0) | - | Ref. (1.0) | - |
|  | 155 to 160 cm | 1.27, [0.97- 1.67] | 0.088 | 1.01, [0.79- 1.28] | 0.967 | 1.02, [0.78- 1.35] | 0.863 |
|  | 150 to 154 cm | 1.73, [1.35- 2.21] | < 0.001 | 1.72, [1.37- 2.16] | < 0.001 | 1.15, [0.89- 1.49] | 0.279 |
|  | 145 to 149 cm | 2.54, [1.93- 3.35] | < 0.001 | 2.36, [1.84- 3.03] | < 0.001 | 1.29, [0.98- 1.69] | 0.071 |
|  | Below 145 cm | 3.17, [2.36- 4.26] | < 0.001 | 2.76, [2.07- 3.69] | < 0.001 | 1.36, [1- 1.86] | 0.052 |
| **Mothers age** | 26 to 35 years^®^ | Ref. (1.0) | - | Ref. (1.0) | - | Ref. (1.0) | - |
|  | 15 to 25 yeas | 1.1, [0.94- 1.3] | 0.247 | 1.38, [1.19- 1.6] | < 0.001 | 1.05, [0.9- 1.23] | 0.515 |
|  | 36 to 49 years | 0.98, [0.79- 1.22] | 0.870 | 1.04, [0.86- 1.27] | 0.657 | 1.31, [0.96- 1.79] | 0.094 |
| **Education** | Illiterate | 1.46, [1.11- 1.92] | 0.007 | 1.57, [1.21- 2.03] | 0.001 | - | - |
|  | Primary | 1.21, [0.9- 1.62] | 0.201 | 1.27, [0.97- 1.68] | 0.085 | - | - |
|  | Secondary | 1.31, [1.04- 1.65] | 0.021 | 1.24, [1- 1.54] | 0.051 | - | - |
|  | Higher | 0.96, [0.75- 1.23] | 0.757 | 0.97, [0.76- 1.25] | 0.836 | - | - |
|  | College^®^ | Ref. (1.0) | - | Ref. (1.0) | - | Ref. (1.0) | - |
| **Children ever born** | Single child^®^ | Ref. (1.0) | - | - | - | - | - |
|  | 2 or 3 children | 1.09, [0.85- 1.39] | 0.497 | 1, [0.79- 1.27] | 0.992 | - | - |
|  | 4 and more children | 1.12, [0.73- 1.72] | 0.608 | 0.9, [0.6- 1.35] | 0.622 | - | - |
| **Breast feeding** | Yes^®^ | - | - | Ref. (1.0) | - | Ref. (1.0) | - |
|  | No | 0.7, [0.59- 0.83] | < 0.001 | 0.81, [0.69- 0.95] | 0.010 | 1.08, [0.92- 1.26] | 0.359 |
| **Child covariates** |  | - | - | - | - | - | - |
| **Child birth order** | First child^®^ | - |  | Ref. (1.0) | - | Ref. (1.0) | - |
|  | Second or third child | 1.25, [1- 1.57] | 0.046 | 1.38, [1.12- 1.71] | 0.002 | - | - |
|  | Four and above | 1.35, [0.88- 2.06] | 0.168 | 2.12, [1.42- 3.17] | < 0.001 | - | - |
| **Age in months** | Less than 13 months^®^ | Ref. (1.0) | - | Ref. (1.0) | - | Ref. (1.0) | - |
|  | 13 to 24 months | 1.3, [1.05- 1.62] | 0.018 | 3.09, [2.46- 3.88] | < 0.001 | 0.41, [0.33- 0.5] | < 0.001 |
|  | 25 to 59 months | 1.83, [1.48- 2.25] | < 0.001 | 2.59, [2.09- 3.21] | < 0.001 | 0.38, [0.32- 0.45] | < 0.001 |
| **Child had cough** | (No) ^®^ | Ref. (1.0) | - | - | - | Ref. (1.0) |  |
|  | Yes | 0.94, [0.78- 1.13] | 0.523 | - | - | 0.81, [0.65- 1] | 0.049 |

1.Confidence interval at 5 % significance level.
