# Supplementary material for: Does maternal overnutrition carry child undernutrition in India?
Source: PLoS One. 2022 Jun 17;17(6):e0265788. doi: 10.1371/journal.pone.0265788 (PMC9205528; doi:10.1371/journal.pone.0265788)
Supplement: S3 Table — (DOCX) [file pone.0265788.s003.docx]

| **S3: Distribution of any form dual burden of malnutrition (overweight or obese mother and underweight/ stunted / wasted child) by familial, maternal and child covariates** | | | | |
| --- | --- | --- | --- | --- |
| **Household covariates** |  | **Overweight/obese mother – undernourished child** | | **Overweight/ obese**  **Mothers, BMI>25** |
|  |  | **Weighted-n** | **(%)** | **Weighted number** |
| **Residence** | Urban | 5259 | 37.2 | 14183 |
|  | Rural | 6044 | 41.3 | 14634 |
| **Wealth quantile** | Poorest | 1046 | 57.4 | 1822 |
|  | Poorer | 1678 | 49.1 | 3417 |
|  | Middle | 2545 | 44.9 | 5673 |
|  | Richer | 3022 | 37.0 | 8171 |
|  | Richest | 3023 | 31.1 | 9732 |
| **Water facilities** | Unimproved water | 10387 | 39.7 | 891 |
|  | Improved | 350 | 39.3 | 26190 |
| **Sanitation facility** | Unimproved sanitation | 6122 | 35.3 | 9753 |
|  | Improved | 4616 | 47.3 | 17328 |
| **Caste** | General or others | 2801 | 34.0 | 8230 |
|  | Other backward class | 5183 | 40.6 | 12672 |
|  | Scheduled castes | 2196 | 43.6 | 5038 |
|  | Scheduled tribes | 582 | 45.9 | 1268 |
| **Religion** | Hindu | 8193 | 38.7 | 21171 |
|  | Sikh | 2454 | 42.6 | 680 |
|  | Christian | 259 | 32.6 | 795 |
|  | Muslims | 217 | 31.9 | 5764 |
|  | Others | 190 | 46.7 | 407 |
| **Maternal covariates** |  |  |  |  |
| **Height** | Above 160 cm | 680 | 26.0 | 2619 |
|  | 155 to 160 cm | 2008 | 30.8 | 6520 |
|  | 150 to 154 cm | 3969 | 38.6 | 10282 |
|  | 145 to 149 cm | 3152 | 47.4 | 6646 |
|  | Below 145 cm | 1504 | 54.7 | 2751 |
| **Mothers age** | 15 to 25 yeas | 3669 | 42.1 | 8710 |
|  | 26 to 35 years | 6576 | 37.4 | 17567 |
|  | 36 to 49 years | 1069 | 42.1 | 2540 |
| **Education** | Illiterate | 2200 | 53.6 | 4107 |
|  | Primary | 1299 | 47.9 | 2710 |
|  | Secondary | 4569 | 39.6 | 11543 |
|  | Higher | 1410 | 33.7 | 4185 |
|  | College | 1835 | 29.3 | 6271 |
| **Children ever born** | Single child | 2491 | 33.8 | 7362 |
|  | 2 or 3 children | 6937 | 39.1 | 17726 |
|  | 4 and more children | 1885 | 50.5 | 3729 |
| **Breast feeding** | Yes | 5136 | 36.5 | 14764 |
|  | No | 6177 | 41.8 | 14053 |
| **work status** | Working | 1722 | 38.6 | 841 |
|  | Not working | 324 | 38.5 | 4455 |
| **Child covariates** |  |  |  |  |
| **Sex of child** | Male | 6149 | 39.9 | 15397 |
|  | Female | 5164 | 38.5 | 13429 |
| **Child birth order** | First child | 3602 | 34.4 | 10483 |
|  | Second or third child | 6075 | 40.1 | 15155 |
|  | Four and above | 1636 | 51.5 | 3180 |
| **Age in months** | Less than 13 months | 1900 | 39.5 | 4807 |
|  | 13 to 24 months | 1987 | 41.8 | 4752 |
|  | 25 to 59 months | 7426 | 38.6 | 19228 |
| **Child had diarrhoea** | No | 10367 | 39.2 | 26448 |
|  | Yes | 937 | 39.9 | 2351 |
| **Child had fever** | No | 9784 | 39.5 | 24797 |
|  | Yes | 1524 | 38.0 | 4010 |
| **Child had cough** | No | 10056 | 39.8 | 25267 |
|  | Yes | 1253 | 35.4 | 3543 |
| **Total** | **-** | **11313** | **39.0** | **28817** |
