# Supplementary material for: Does maternal overnutrition carry child undernutrition in India?
Source: PLoS One. 2022 Jun 17;17(6):e0265788. doi: 10.1371/journal.pone.0265788 (PMC9205528; doi:10.1371/journal.pone.0265788)
Supplement: S4 Table — (DOCX) [file pone.0265788.s004.docx]

| **S4: logistic regression results of any form of dual burden of malnutrition (overweight or obese mother and underweight/ stunted/wasted child) by familial, maternal and child covariates** | | | | | |
| --- | --- | --- | --- | --- | --- |
| **Household covariates** |  | **Overweight or obese mother and undernourished (underweight/stunted/wasted) child pairs** | | | |
|  |  | **ORs [CI]^1^** | ***p-value*** | **aORs^2^ [CI]** | ***p-value*** |
| **Residence** | Urban^®^ | Reference (1.0) | - |  |  |
|  | Rural | 1.19, [1.10 - 1.29] | <0.001 | 0.87, [0.79 - 0.96] | 0.004 |
| **Wealth quantile** | Poorest | 2.99, [2.56 - 3.48] | <0.001 | 1.73, [1.39 - 2.16] | <0.001 |
|  | Poorer | 2.14, [1.89 - 2.43] | <0.001 | 1.43, [1.20 - 1.70] | <0.001 |
|  | Middle | 1.81, [1.61 - 2.03] | <0.001 | 1.35, [1.17 - 1.57] | <0.001 |
|  | Richer | 1.30, [1.17 - 1.45] | <0.001 | 1.07, [0.95 - 1.22] | 0.265 |
|  | Richest^®^ | Reference (1.0) | - |  |  |
| **Water facilities** | Unimproved water | 0.99, [0.79 - 1.23] | 0.900 |  |  |
|  | Improved^®^ | Reference (1.0) | - |  |  |
| **Sanitation facility** | Unimproved sanitation | 1.64, [1.51 - 1.79] | <0.001 | 1.06, [0.95 - 1.18] | 0.286 |
|  | Improved ^®^ | Reference (1.0) |  |  |  |
| **Caste** | General or others^®^ | Reference (1.0) | - |  |  |
|  | Other backward class | 1.33, [1.20 - 1.47] | <0.001 | 1.21, [1.09 - 1.35] | <0.001 |
|  | Scheduled castes | 1.50, [1.20 - 1.47] | <0.001 | 1.18, [1.04 - 1.35] | 0.013 |
|  | Scheduled tribes | 1.64, [1.39 - 1.95] | <0.001 | 1.19, [0.98 - 1.44] | 0.082 |
| **Religion** | Hindu^®^ | Reference (1.0) | - |  |  |
|  | Sikh | 0.74, [0.59 - 0.93] | 0.008 | 1.04, [0.83 - 1.31] | 0.735 |
|  | Christian | 0.77, [0.60 - 0.97] | 0.029 | 0.93, [0.73 - 1.19] | 0.577 |
|  | Muslims | 1.17, [1.06 - 1.30] | 0.003 | 1.07, [0.94 - 1.21] | 0.337 |
|  | Others | 1.39, [0.93 - 2.06] | 0.106 | 1.31, [0.84 - 2.04] | 0.238 |
| **Maternal covariates** |  |  |  |  |  |
| **Height** | Above 160 cm^®^ | Reference (1.0) | - |  |  |
|  | 155 to 160 cm | 1.27, [1.08 - 1.49] | 0.003 | 1.10, [1.01 - 1.41] | 0.036 |
|  | 150 to 154 cm | 1.79, [1.54 - 2.09] | <0.001 | 1.62, [1.38 - 1.90] | <0.001 |
|  | 145 to 149 cm | 2.57, [2.20 - 3.02] | <0.001 | 2.20, [1.86 - 2.61] | <0.001 |
|  | Below 145 cm | 3.44, [2.81 - 4.21] | <0.001 | 2.64, [2.13 - 3.27] | <0.001 |
| **Mothers age** | 26 to 35 years^®^ | Reference (1.0) | - |  |  |
|  | 15 to 25 yeas | 1.22, [1.12 - 1.32] | <0.001 | 1.25, [1.13 - 1.39] | <0.001 |
|  | 36 to 49 years | 1.22, [1.07 - 1.39] | 0.004 | 1.03, [0.89 - 1.20] | 0.659 |
| **Education** | Illiterate | 2.79, [2.45 - 3.18] | <0.001 | 1.55, [1.30 - 1.85] | <0.001 |
|  | Primary | 2.23, [1.92 - 2.59] | <0.001 | 1.42, [1.18 - 1.70] | <0.001 |
|  | Secondary | 1.58, [1.41 - 1.78] | <0.001 | 1.20, [1.06 - 1.37] | <0.001 |
|  | Higher | 1.23, [1.06 - 1.42] | <0.005 | 1.10, [0.94 - 1.28] | 0.243 |
|  | College^®^ | Reference (1.0) |  |  |  |
| **Children ever born** | Single child^®^ | Reference (1.0) | - |  |  |
|  | 2 or 3 children | 1.26, [1.14 - 1.39] | <0.001 | 0.97, [0.83 - 1.14] | 0.736 |
|  | 4 and more children | 2.00, [1.77 - 2.25] | <0.001 | 0.94, [0.71 - 1.24] | 0.660 |
| **Breast feeding** | Yes^®^ | Reference (1.0) | - |  |  |
|  | No | 0.80, [0.74 - 0.87] | <0.001 | 0.85, [0.77 - 0.93] | <0.001 |
| **work status** | Working^®^ | Reference (1.0) | - |  |  |
|  | Not working | 1.01, [0.81 - 1.24] | 0.962 |  |  |
| **Child covariates** |  |  |  |  |  |
| **Sex of child** | Male^®^ | 1.06, [0.98 - 1.16] | 0.150 |  |  |
|  | Female | Reference (1.0) | - |  |  |
| **Child birth order** | First child^®^ | Reference (1.0) | - |  |  |
|  | Second or third child | 1.28, [1.17 - 1.39] | <0.001 | 1.32, [1.16 - 1.52] | <0.001 |
|  | Four and above | 2.03, [1.81 - 2.27] | <0.001 | 1.59, [1.21 - 2.09] | <0.001 |
| **Age in months** | Less than 13 months^®^ | Reference (1.0) | - |  |  |
|  | 13 to 24 months | 1.10, [0.97 - 1.25] | 0.148 |  |  |
|  | 25 to 59 months | 0.96, [0.87 - 1.06] | 0.421 |  |  |
| **Child had diarrhoea** | No^®^ | Reference (1.0) | - |  |  |
|  | Yes | 1.03, [0.91 - 1.16] | 0.658 |  |  |
| **Child had fever** | No ^®^ | Reference (1.0) | - |  |  |
|  | Yes | 0.94, [0.85 - 1.04] | 0.251 |  |  |
| **Child had cough** | (No) ^®^ | Reference (1.0) | - |  |  |
|  | Yes | 0.83, [0.75 - 0.92] | <0.001 | 0.80, [0.71 - 0.89] | <0.001 |

1.Odds ratios and confidence interval at 5 % significance level of bivariate regression models

2. Odds ratios in multivariable model after adjusting for the variables which were significant at 5 % level in bivariate models.
